# Church of England Sanitary Association

**Published:** 1893-03-11

**Authors:** 


### Church of England Sanitary Association.

Lord Brassey, presiding at the March monthly meeting of the Church of England Sanitary Association at the Church House, Westminster, said that this society had been founded to secure for all healthy dwellings, pure air, pure water, unadulterated food, and the greatest possible immunity from infectious diseases, and, generally, to cultivate such conditions of life as to ensure for the individual the highest possible standard of health. While conscious of the objections to the multiplication of societies, the promoters of this society submitted that its formation was not merely justifiable but imperatively demanded, because the extent of preventible illness and mortality, and the appalling amount of sorrow, the degeneracy of race, and the vast pecuniary loss consequent thereupon in this country constituted a national disgrace; because also the parochial system of the Church afforded an ideal, and indeed the only competent organisation for carrying on the work of sanitary reform on an adequate scale. Mr. Ernest Hart, editor of the *British Medical Journal*, in the course of a paper upon "The Work of the Clergy in Respect of Sanitation," said that the main objects of sanitary science were (a) purity of air, soil, water and food; (b) isolation of infectious diseases and disinfection; and (c) thorough personal hygiene. The fact that the Hebrew clergy, living still under the old Mosaic dispensation, taught as part of their official religious duty the observance of sanitary laws, had resulted in the Jews being preserved to an astonishing extent throughout all the great epidemics of past and recent times. Indeed, their preservation from disease had been over and over again the very cause of their persecution, and they were still the healthiest people in Europe. The death-rate in the East End of London was now 30 per cent. below that among the Gentiles. The black pest and other plagues of mediæval times, and our modern zymotic diseases with their death roll of millions, were to be traced to the neglect of the laws of health which came after the new religionists who taught that the sanitary laws of Moses were only dead ceremonial. The eminent sanitarian concluded by urging the clergy of the Church of England to adopt as a strict ecclesiastical mandate the duty of teaching their people to keep earth, water, food, and air absolutely pure. He also expressed an earnest hope that the Church organisations would be used for the purpose of promoting sanitary knowledge, and of forming sanitary committees in every parish in the land. A resolution was then moved by Dr. Farquharson, M.P., seconded by Dr. Norman Kerr, and carried unanimously, congratulating the Church upon having taken up sanitation as a special feature of her work. Votes of thanks to Mr. Ernest Hart for his paper, and to Lord Brassey for presiding, were spoken to by General Lowry, C.B., Canon Barker, the Rev. T. B. Paynter, Mr. J. Furley, and the Rev. F. Lawrence (hon. sec.)

